# Immunosenescence in *Caenorhabditis elegans*

**DOI:** 10.1186/s12979-022-00314-8

**Published:** 2022-11-15

**Authors:** Sieun S. Kim, Jooyeon Sohn, Seung-Jae V. Lee

**Affiliations:** Department of Biological Sciences, Korea Advanced Institute of Science and Technology, 291 Daehak-ro, Yuseong-gu, Daejeon, 34141 South Korea

**Keywords:** Immunosenescence, *Caenorhabditis elegans*, Aging, MAPK, SKN-1/NRF, Insulin/IGF-1

## Abstract

Immunosenescence is an age-dependent decline in immune functions and hallmark of aging in diverse species, ranging from invertebrates to mammals. However, identifying the factors responsible for immunosenescence is challenging because of the complexity of immune systems and aging in mammals. The roundworm *Caenorhabditis elegans* is suitable for understanding immunosenescence because of its simple immune system and rapid aging process. In this review, we discuss the advances in our understanding of immunosenescence in *C. elegans*. PMK-1/p38 mitogen-activated protein kinase (MAPK), SKN-1/NRF, and ZIP-10/bZIP transcription factor regulate immunosenescence through p38 MAPK and insulin/IGF-1 signaling pathways. Because these factors and pathways are evolutionarily conserved, the findings discussed in this review may help understand the mechanisms underlying immunosenescence and develop new treatment therapy for immunosenescence in humans.

## Background

Aging has been regarded as a universal and inevitable biological process in almost all living organisms. One of the hallmarks of aging is immunosenescence, a gradual age-dependent decline in immune functions. Although the contexts of immunosenescence in humans are controversial [1, 2], decreases in the efficiency of adaptive immunity and innate immunity, and in immune responses to vaccination have been reported [3, 4, 5]. For example, the elderly are vulnerable to infection with novel pathogens, such as SARS-CoV-2, although the protection effect of vaccine on older adults are the same as that on younger people [2, 6]. Several recent studies have helped us understand the molecular mechanisms driving immunosenescence and develop strategies to delay or even reverse immunosenescence. Although the hallmarks of immunosenescence have been proposed [5, 7], the causal factors for immunosenescence remain poorly understood.

Because of its short lifespan and genetic tractability, the roundworm *Caenorhabditis elegans* is an excellent model for studying basic animal physiology, including aging, stress response, immunity, and immunosenescence [8, 9, 10]. Various differences and similarities exist between immunity and immunosenescence in *C. elegans* and those in mammals. *C. elegans* lacks an adaptive immune system, specialized immune cells, and NF-κB signaling, which are crucial for mammalian immunity [10, 11]. However, *C. elegans* has an innate immune system regulated by evolutionarily conserved signaling pathways, including p38 MAPK, insulin/IGF-1 signaling (IIS) and DBL-1/TGF- β pathways [10, 11, 12, 13, 14, 15]. *C. elegans* displays an age-dependent decline in immunity, as demonstrated by using *Pseudomonas aeruginosa*, opportunistic pathogenic bacteria to humans, as a model pathogen [16, 17, 18, 19]. The majority of mammalian immunosenescence research has focused on the aging of specialized immune cells. In contrast, studies on *C. elegans* immunosenescence have mostly reported age-dependent impairments in immune organ functions and signaling pathways. Nevertheless, the immune systems in mammals are very complex, and therefore studies using *C. elegans* can provide insights into fundamental mechanisms underlying immunosenescence.

In this short review, we discuss findings regarding immunosenescence in *C. elegans*, focusing on three papers that functionally characterized the genetic factors that contribute to immunosenescence [16, 17, 19]. These papers demonstrated that PMK-1/p38 mitogen-activated protein kinase (MAPK), SKN-1/NRF, and DAF-2/insulin/IGF-1 receptor, established regulators of aging, immunity, and stress responses [12, 14, 17, 20, 21, 22], affect immunosenescence via acting in the p38 MAPK and IIS pathways. Because these signaling pathways are evolutionarily conserved from *C. elegans* to mammals, the findings we discuss here will lead to the development of therapeutic strategies for delaying or even reversing immunosenescence in humans.

## Main text

### Age-dependent decline in p38 MAPK signaling contributes to immunosenescence in *C. elegans*

Mammalian p38 MAPK is crucial for immunity and inflammatory responses [23, 24]. PMK-1, a *C. elegans* ortholog of p38 MAPK, has been established as a key regulator of innate immunity, which upregulates transcription factors, including ATF-7/activating transcription factor 7 and SKN-1/NRF [12, 17, 20]. Therefore, investigating the role of PMK-1/p38 MAPK in immunosenescence was an intriguing and vital question to address.

**Fig. 1:**
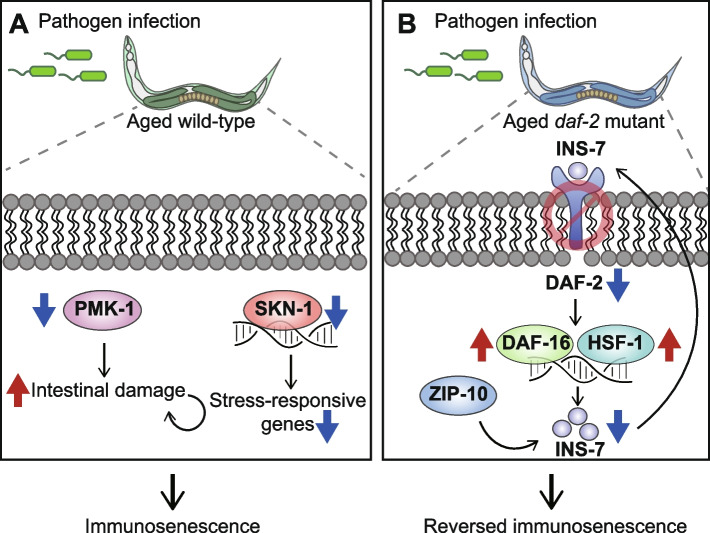
Factors and pathways that regulate immunosenescence in *Caenorhabditis elegans*. **A** Upon pathogen infection of aged wild-type *C. elegans*, downregulation of PMK-1/p38 mitogen-activated protein kinase (MAPK) leads to intestinal damage and immunosenescence. In addition, downregulation of SKN-1/NRF transcription factor decreases the expression of stress-responsive genes and contributes to immunosenescence. **B** Aged *daf-2* mutant *C. elegans* displays reversed immunosenescence. Genetic inhibition of DAF-2/insulin/IGF-1 receptor upregulates DAF-16/FOXO and HSF-1/heat-shock transcription factor 1. This leads to downregulation of INS-7/insulin-like peptide, which is also regulated by the ZIP-10/bZIP transcription factor, resulting in further reduction of DAF-2 signaling, and reverses immunosenescence

Youngman et al. reported that the age-dependent decline of PMK-1 activity underlies immunosenescence by accelerating the deterioration of the intestine, a main immune organ in *C. elegans* [16]. The authors showed that PMK-1/p38 MAPK is downregulated during aging at the transcriptional and translational levels. The mRNA levels of PMK-1/p38 MAPK targets decrease with age. The authors then performed survival assays by infecting animals with *P. aeruginosa* PA14 strain at the juvenile L4 larval stage, and day 3 (young), day 6 (middle age), and day 9 (old) of adulthoods, and determined the role of PMK-1/p38 MAPK in immunosenescence. The effects of *pmk-1* mutations on impaired pathogen resistance decrease in aged animals depleted of PMK-1. Therefore, the authors concluded that the reduced levels of PMK-1/p38 MAPK in aged *C. elegans* contribute to age-dependent impairment of immune responses (Fig. 1A). The authors then showed that *pmk-1* mutations induce the accumulation of bacteria in the intestine, leading to intestinal damage. In conclusion, gradual downregulation of PMK-1/p38 MAPK promotes intestinal aging and impairs immune function, accumulating pathogenic bacteria in the gut, which induces damage in the intestine. This study highlights the role of PMK-1/p38 MAPK, which protects animals from intestinal tissue damage in young age, and its age-dependent downregulation leads to immunosenescence via a vicious circle of damage and pathogen infection in the intestine.

### SKN-1/NRF prevents immunosenescence in young *C. elegans*

The bacterial virulence factors, such as bacterial toxins, often induce damage in the host intestine by counteracting the host antimicrobial defense mechanisms, and several stress regulators, including the components in IIS, regulate immunity [25, 26]. The transcription factor SKN-1/NRF, which regulates oxidative and xenobiotic stress responses, longevity, and immunity, acts in both p38 MAPK and IIS pathways [14, 21, 27]. However, the role of SKN-1/NRF in immunosenescence was unknown.

Papp et al. reported that SKN-1/NRF is required for normal immunity against gram-negative *P. aeruginosa* PA14 and gram-positive *Enterococcus faecalis* [17]. PA14 infection increases the nuclear localization of SKN-1/NRF through Toll/IL-1 Receptor domain protein/TIR-1 and PMK-1/p38 MAPK and subsequently increases the expression of SKN-1/NRF target genes, γ glutamylcysteine synthetase 1 (*gcs-1*) and glutathione S-transferase 4 (*gst-4*). They also showed an age-dependent decrease in the expression of SKN-1/NRF target genes upon PA14 infection. The authors experimentally evaluated the relationship between SKN-1/NRF activity and immunosenescence and found that SKN-1/NRF target gene expression decreases with age (Fig. 1A). In addition, the survival of day 4 wild-type animals on PA14 is similar to that of day 1 *skn-1* mutant animals. These results indicate that a decline in SKN-1/NRF function contributes to immunosenescence. Furthermore, SKN-1/NRF is required to enhance pathogen resistance mediated by reduced IIS or oxidative preconditioning. However, the hyperactivation of SKN-1/NRF by the genetic inhibition of WDR-23, a direct repressor of SKN-1, impairs pathogen resistance. Thus, the optimal activity of SKN-1/NRF appears crucial for maintaining proper immune responses, consistent with our recent study on the proper regulation of SKN-1/NRF for enhanced immunity and healthy longevity [28]. Overall, this study identifies SKN-1/NRF as a critical factor that contributes to preventing premature immunosenescence in *C. elegans*.

### Reduction of IIS can reverse immunosenescence in *C. elegans* through a positive feedback loop

Increased innate immunity at a young age is a general feature in various long-lived *C. elegans* mutants [13, 14, 25, 29, 30, 31]. Thus, interventions that alter longevity may have causal effects on immunosenescence, but whether and how these genetic factors affect immunosenescence remained elusive.

Our study showed that DAF-2/insulin/IGF-1 receptor contributes to immunosenescence by inducing INS-7/insulin-like peptide 7 through DAF-16/FOXO, HSF-1/heat-shock transcription factor 1, and ZIP-10/bZIP transcription factors [19]. We aimed to identify longevity-promoting interventions that modulate immunosenescence by using five long-lived mutants: insulin/IGF-1 receptor-defective *daf-2*, sensory neuron-compromised *osm-5*, dietary restricted *eat-2*, mitochondrial respiration-impaired *isp-1*, and germline-deficient *glp-1* mutants. Surprisingly, we found that day 9 (old) *daf*-2 mutants displayed enhanced pathogen resistance against PA14 compared to day 1 (young) *daf*-2 mutants that are more resistant to PA14 than wild-type animals. The effect is, at least in part, independent of PA14 intake or clearance. These results indicate that genetic inhibition of DAF-2, which substantially increases lifespan, stress resistance and immunity [13, 14, 32, 33, 34], can cause immune rejuvenation (Fig. 1 B).

DAF-16/FOXO, SKN-1/NRF, and HSF-1 are key transcription factors acting downstream of the DAF-2/insulin/IGF-1 receptor [13, 14], and are crucial for longevity and immunity [17, 25, 35, 36]. We showed that DAF-16/FOXO and HSF-1 are required for enhanced immunocompetence in old *daf-2* mutants compared with young *daf-2* mutants, whereas SKN-1/NRF is not essential. We also found that depletion of PMK-1/p38 MAPK, a major immune-regulatory factor whose downregulation contributes to immunosenescence [16], is not required for the further enhanced immunocompetence of aged *daf-2* mutants. Thus, DAF-16/FOXO and HSF-1 are key factors that mediate immune rejuvenation in *daf-2* mutants.

We searched for genes that mediate enhanced immunocompetence in old *daf-2* mutants by analyzing RNA-seq data using day 1 (young) and 9 (old) adult wild-type and *daf-2* mutant animals. We found that *ins-7*, an agonistic insulin-like peptide [37, 38, 39], is downregulated during aging in *daf-2* mutants by activating DAF-16/FOXO and HSF-1. Mutations in *ins-7* are sufficient to enhance pathogen resistance in old animals. We concluded that the downregulation of *ins-7* contributes to delayed immunosenescence in *daf-2* mutants.

We then focused on the ZIP-10/bZIP transcription factor, whose expression increases with age in wild-type animals but decreases in *daf-2* mutants, similar to that of *ins-7*. ZIP-10/bZIP transcription factor plays a role in the defense response against pathogens in plants [40]. We showed that genetic inhibition of ZIP-10 suppresses age-dependent upregulation of *ins-7* and increases PA14 resistance in day 9 adult animals. Therefore, downregulation of ZIP-10/bZIP transcription factor in aged *daf-2* mutants prevents immunosenescence by increasing the activities of DAF-16/FOXO and HSF-1 by decreasing the levels of INS-7 (Fig. 1 B). This study provides clues for reversing immunosenescence by revealing the role of an immunity-regulating positive feedback loop in IIS in aged *C. elegans*.

## Conclusions

Many previous studies have suggested that age-dependent accumulation of PA14 along with the extensive deterioration of the intestine, a critical immune organ in *C. elegans* and mammals, is the primary cause of immunosenescence [12, 41, 42, 43]. Consistent with the reports, the two studies discussed here have demonstrated that age-dependent decline in critical immune factors, PMK-1/p38 MAPK and SKN-1/NRF, in the intestine, are responsible for immunosenescence [16, 17]. While increased colonization of PA14 in the intestine in aged *C. elegans* is the main cause of immunosenescence*,* our work suggests that genetic inhibition of *daf-2* can further increase immunocompetence in old age, even under conditions with PA14 accumulation [19]. The abovementioned data highlight the complexity of the links between immunity and aging, suggesting that the relationship is context dependent.

Because studies regarding immunosenescence in *C. elegans* are limited, identifying novel factors that delay or reverse immune aging is required to further understand the relationship between immunity and aging. In *C. elegans*, several signaling pathways, including autophagy, unfolded protein response, and DBL-1/TGF- β pathway, regulate immunity and aging, in addition to p38 MAPK and IIS pathways that are discussed here [10, 11, 15, 44]. Therefore, studying the roles of all these pathways in the context of age-dependent changes in immunity will provide additional mechanistic insights into immunosenescence. In addition, as immunosenescence studies in *C. elegans* have been focused on antibacterial immunity, research regarding age-dependent changes in immune responses against virus and fungi will be crucial.

The studies discussed here identified and characterized genetic factors that regulate immunosenescence in *C. elegans*. In addition, our study provides information for potential strategies to reverse immune aging [19]. As many factors that regulate aging and immunity are evolutionarily conserved from *C. elegans* to mammals, factors and pathways discussed here may also regulate immunosenescence in humans. Consistent with this possibility, p38 MAPK and insulin/IGF-1 signaling contributes to immunosenescence in mammals [5, 45]. Thus, further research is required to unravel the molecular mechanism of immunosenescence in humans to improve treatment therapy of age-related pathologies.
